# Complete response of extramedullary relapse in breast of acute T lymphoblastic leukemia after bone marrow transplantation to chemoradiotherapy: a case report and literature review

**DOI:** 10.1186/s12885-016-2910-0

**Published:** 2016-11-09

**Authors:** Bailong Liu, Bin Liu, Xu Wang, Liang Guo, Xiaoliang Liu, Wei Han, Lihua Dong, Min Liu

**Affiliations:** 1 Department of Radiation Oncology, The First Hospital, Jilin University, 71 Xinmin Street, Changchun, 130021 China; 2 Department of Hand Surgery, The First Hospital, Jilin University, 71 Xinmin Street, Changchun, 130021 China; 3 Cancer Center, The First Hospital, Jilin University, 71 Xinmin Street, Changchun, 130021 China; 4 Department of Pathology, The First Hospital, Jilin University, 71 Xinmin Street, Changchun, 130021 China

**Keywords:** Radiotherapy, Extramedullary relapse, Breast, Acute lymphoblastic leukemia, Transplantation, Case report

## Abstract

**Background:**

Relapse of acute lymphoblastic leukemia (ALL) occurring in the breast after allografting is extremely rare, with only 22 reported cases in the literature thus far. Further, the lack of a systemic analysis provides little information about this entity. We present a case of isolated extramedullary relapse from acute T lymphoblastic leukemia (ATLL) after allogeneic hematopoietic stem cell transplantation (HSCT).

**Case presentation:**

A 32-year-old Chinese woman diagnosed with ATLL with myeloid antigen expression received HSCT from her human leukocyte antigen (HLA)-matched sister and presented with two lesions in her right breast 6 months later. Pathology investigation revealed breast relapse, with complete remission on the basis of bone marrow findings. Combined modality treatment including chemotherapy and local radiotherapy helped achieve complete remission with mild side effects.

**Conclusion:**

The findings from this case indicate that the breast is a potentially involved extramedullary site of relapse for ALL patients after HSCT. In the case of a newly developed breast lump in such patients, clinicians consider local relapse even if the bone marrow findings indicate remission. Combined modality treatment will contribute to better local control and improve prognosis.

## Background

Relapse of acute lymphoblastic leukemia (ALL) occurring in the breast after allografting is extremely rare, with only 22 reported cases in the literature thus far [1–8]. Isolated extramedullary relapse (IEMR) in the so-called sanctuary sites after hematopoietic stem cell transplantation (HSCT) is rarely encountered in clinical practice, especially in the breast [4]. Given the paucity of data surrounding this phenomenon, the optimal treatment for relapse of ALL in the breast has not been identified yet. Systemic chemotherapy is generally used. Herein, we describe a rather rare case of IEMR in the breast after HSCT for acute T lymphoblastic leukemia (ATLL) in which combined modality treatment contribute to better local control and improve prognosis.

## Case presentation

**Fig. 1:**
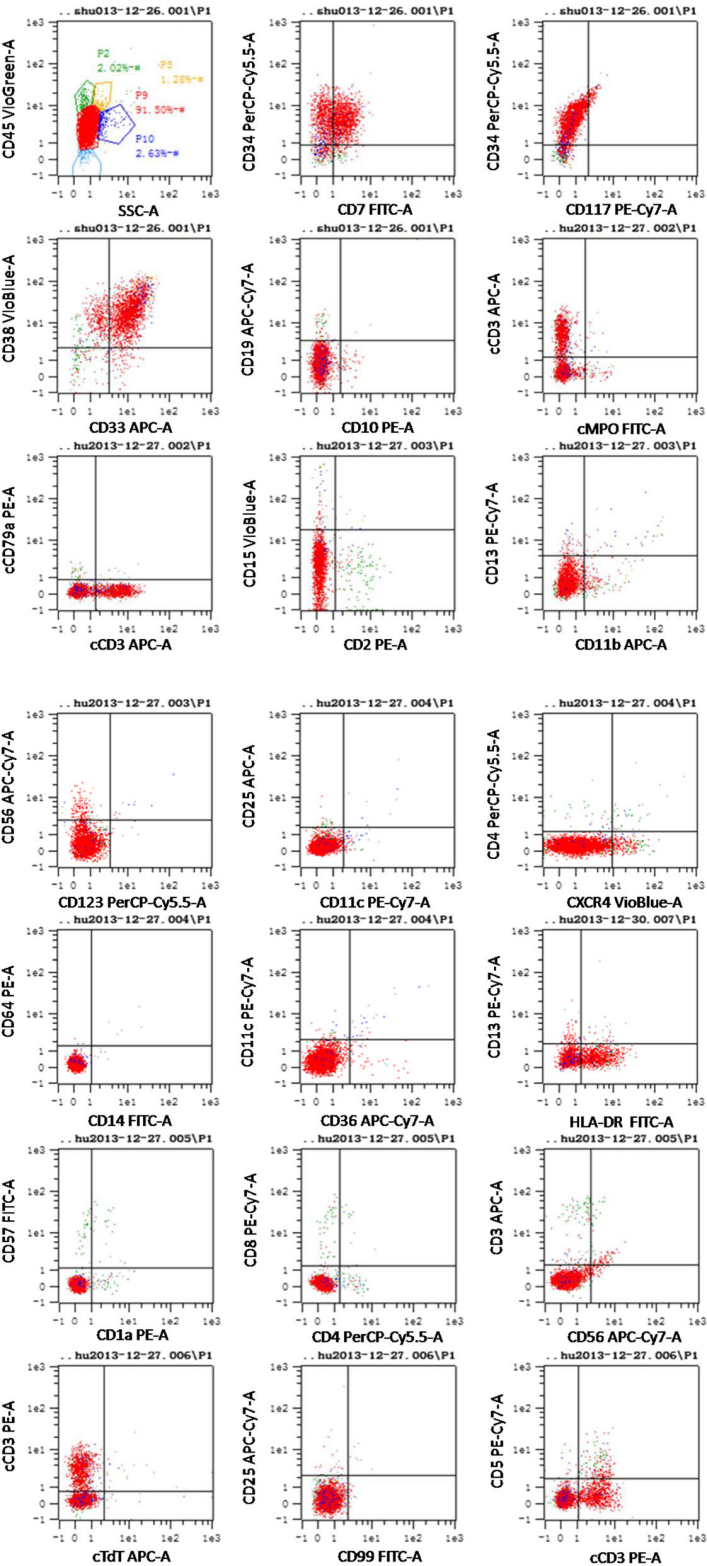
Flow cytometry results of the marrow aspirate at the first visit

In December 2013, a 32-year-old Chinese woman presented with a 1-month history of intermittent fever. Her family, psychosocial and past history was unremarkable. On physical examination, pallor was significant. Neither lymphadenopathy nor hepatosplenomegaly could be detected. The complete blood cell count examination demonstrated significantly increased leukocyte (125 × 10^9^ /L) and decreased hemoglobin (71 g/L) and platelet (17 × 10^9^ /L) values. The subsequent peripheral blood smears and bone marrow aspirate showed blast cell infiltration of 91 and 89.5 %, respectively. Flow cytometry analysis of the bone marrow aspirate revealed that the blast cells (P9) accounted for 91.50 % of the cell population, which predominantly expressed CD34, CD7, CD33, CD38dim, and HLA-DR; partly expressed cCD3; seldom expressed cMPO, CD56, and CD13; and did not express CD117, cCD79a, CD10, CD19, CD2, CD3, CD99, CD123, CD11b, CD15, CD14, CD64, CD4, CD11c, CD25, CD36, CXCR4, cTdT, CD5, CD57, CD1a, and CD8 (Fig. 1). Therefore, the patient was diagnosed with ATLL with myeloid antigen expression. Fusion gene screening revealed *SET-CAN* positivity. Induction chemotherapy using a VDCLP (vincristine, daunorubicin, cyclophosphamide, L-asparaginase and prednisone) regimen failed to achieve remission. The treatment was switched to a CAM (cyclophosphamide, cytarabine and 6-mercaptopurine) regimen, which induced complete remission (CR). Consolidation chemotherapy including one cycle of CAM and two cycles of HD-MTX (high dose methotrexate) was administered during which intrathecal chemotherapy was provided 12 times.

In May 2014, the patient received allogeneic HSCT from her human leukocyte antigen (HLA)-matched sister. The conditioning regimen administered was modified BU/CY (busulfan/cyclophosphamide). CSA (cyclosporin), CellCept, and short-term methotrexate (MTX) were used to prevent graft-versus-host disease (GVHD). We administered an infusion of 3.2 × 10^6^/kg CD34+ cells and 11.26 × 10^8^/kg mononuclear cells. Megakaryocyte and granulocyte engraftment occurred at day +9 and +13, respectively. Bone marrow evaluation at day +14, +28, +42, +60, +90 indicated CR. Additionally, flow cytometry did not indicate minimal residual disease. The patient had 100 % donor chimerism. After HSCT, 4 cycles of intrathecal chemotherapy were conducted and cerebrospinal fluid examination results were unremarkable. The post-transplant course was uneventful, without GVHD.

**Fig. 2:**
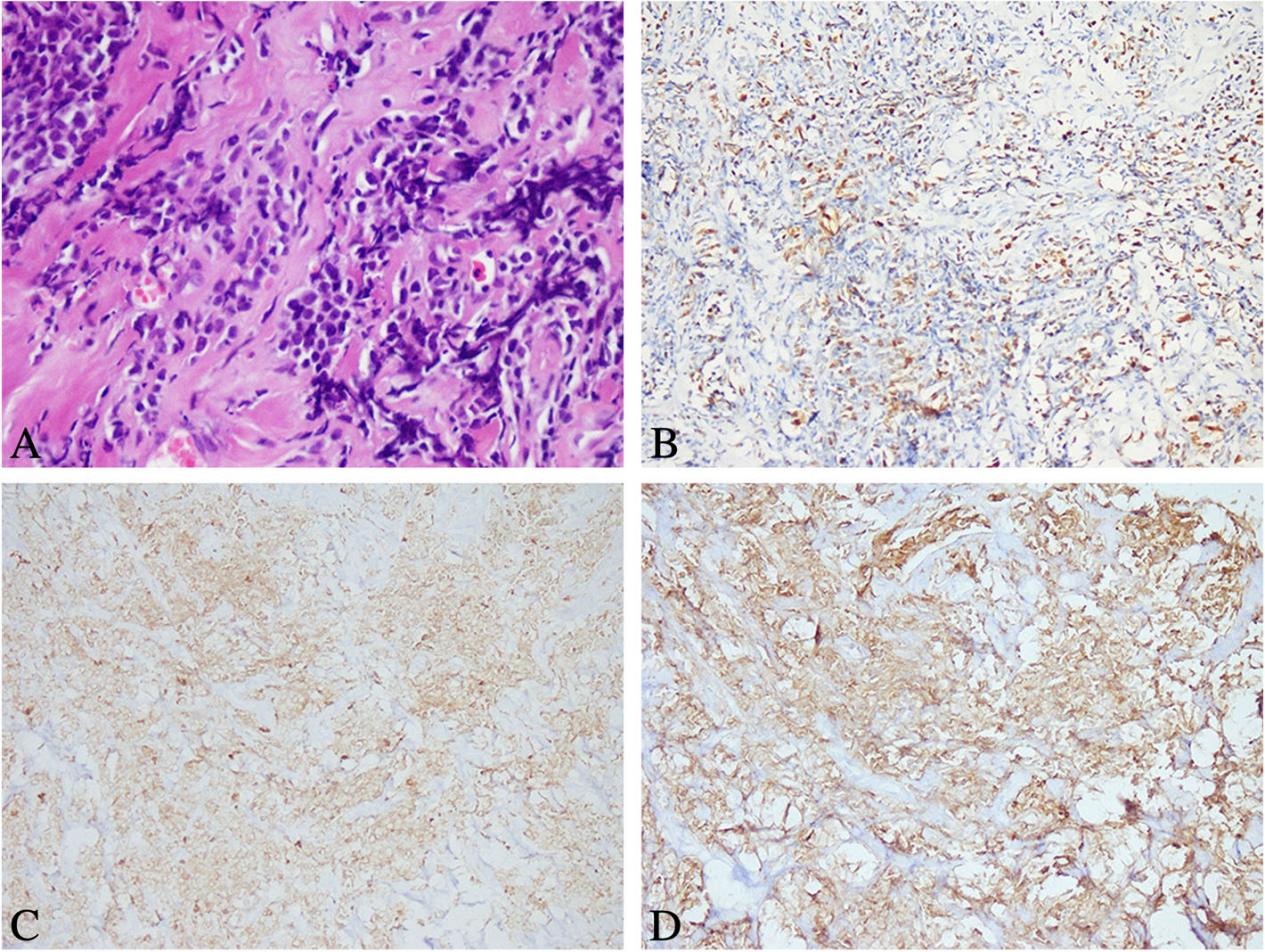
Histologic and immunohistochemistry results of the biopsy of the right breast tumor. **a** The biopsy of the large lump indicated the presence of a small round cell tumor (HE staining, original magnification, 400×). **b** The neoplasm exhibited a high Ki-67 proliferation index (60 %, original magnification, 200×) and stained positive for CD3 (**c**, original magnification, 200×) and CD56 (**d**, original magnification, 200×)

**Fig. 3:**
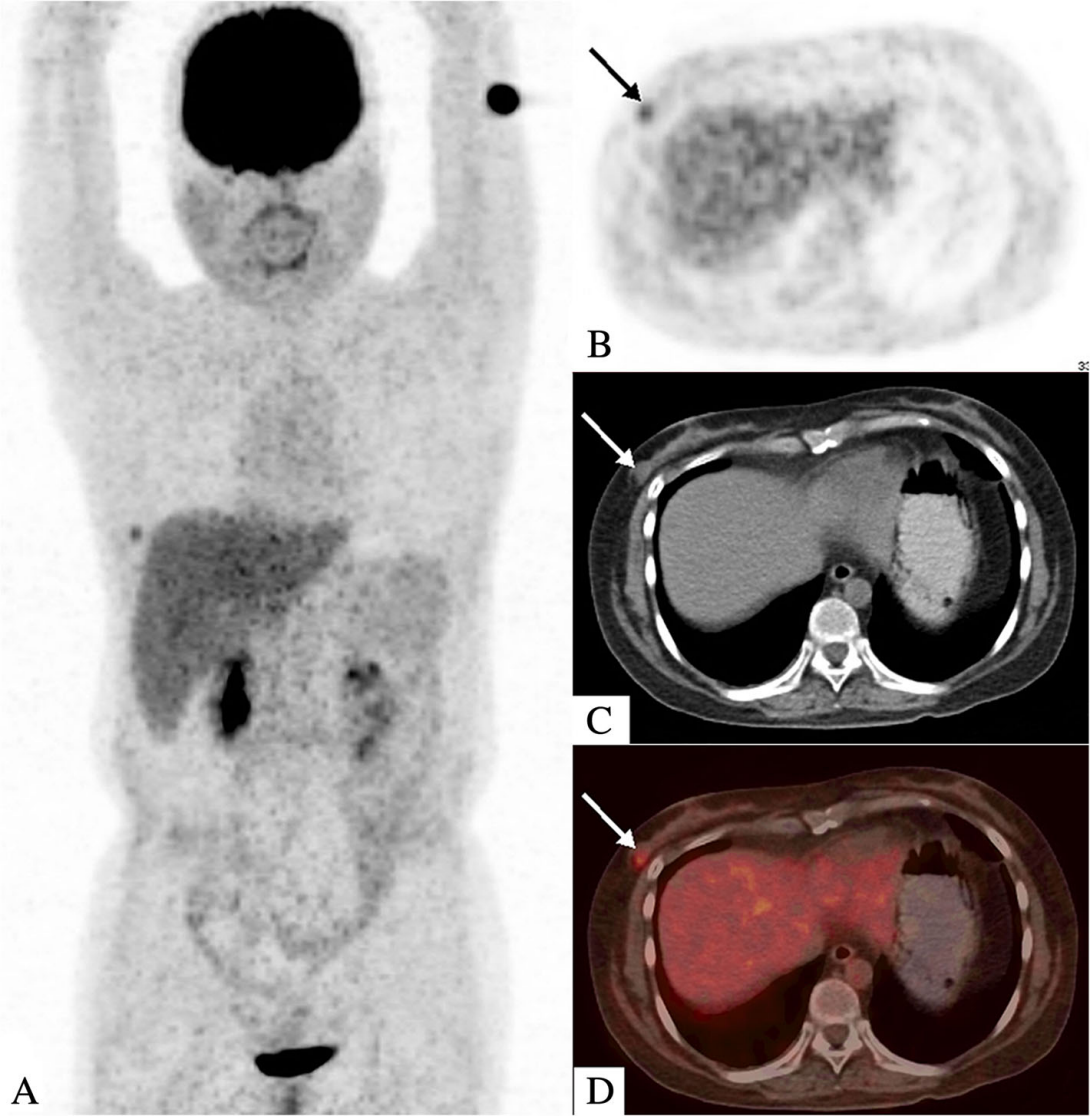
Whole-body positron-emission tomography/computed tomography (PET/CT) findings. **a** The PET/CT scan demonstrated a hypermetabolic nodule in right breast and no focal uptake in other sites. The 18 F-FDG-avid nodule was located in the outer upper quadrant of the right breast with a SUV of 4.6 (**b**, PET images; **c**, CT images; **d**, fused PET/CT images)

**Fig. 4:**
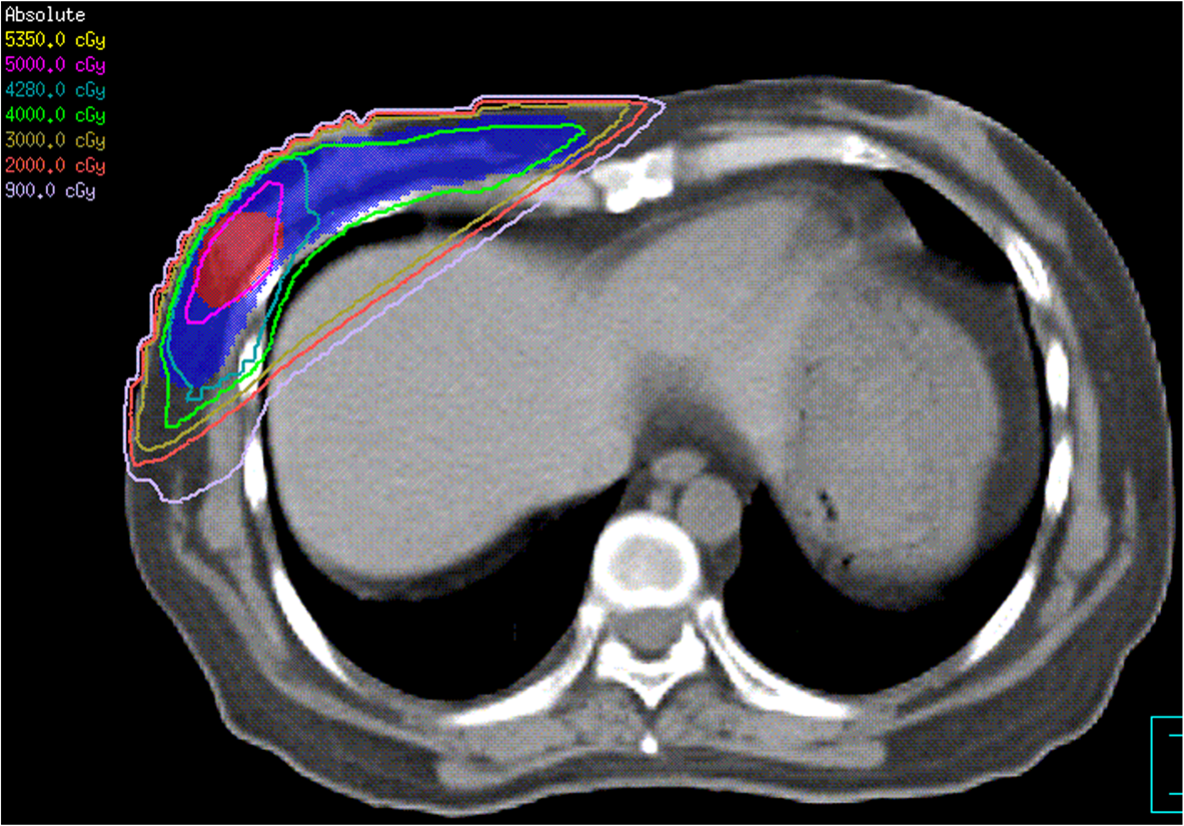
The dose distribution of the simple intensity-modulated radiotherapy

**Fig. 5:**
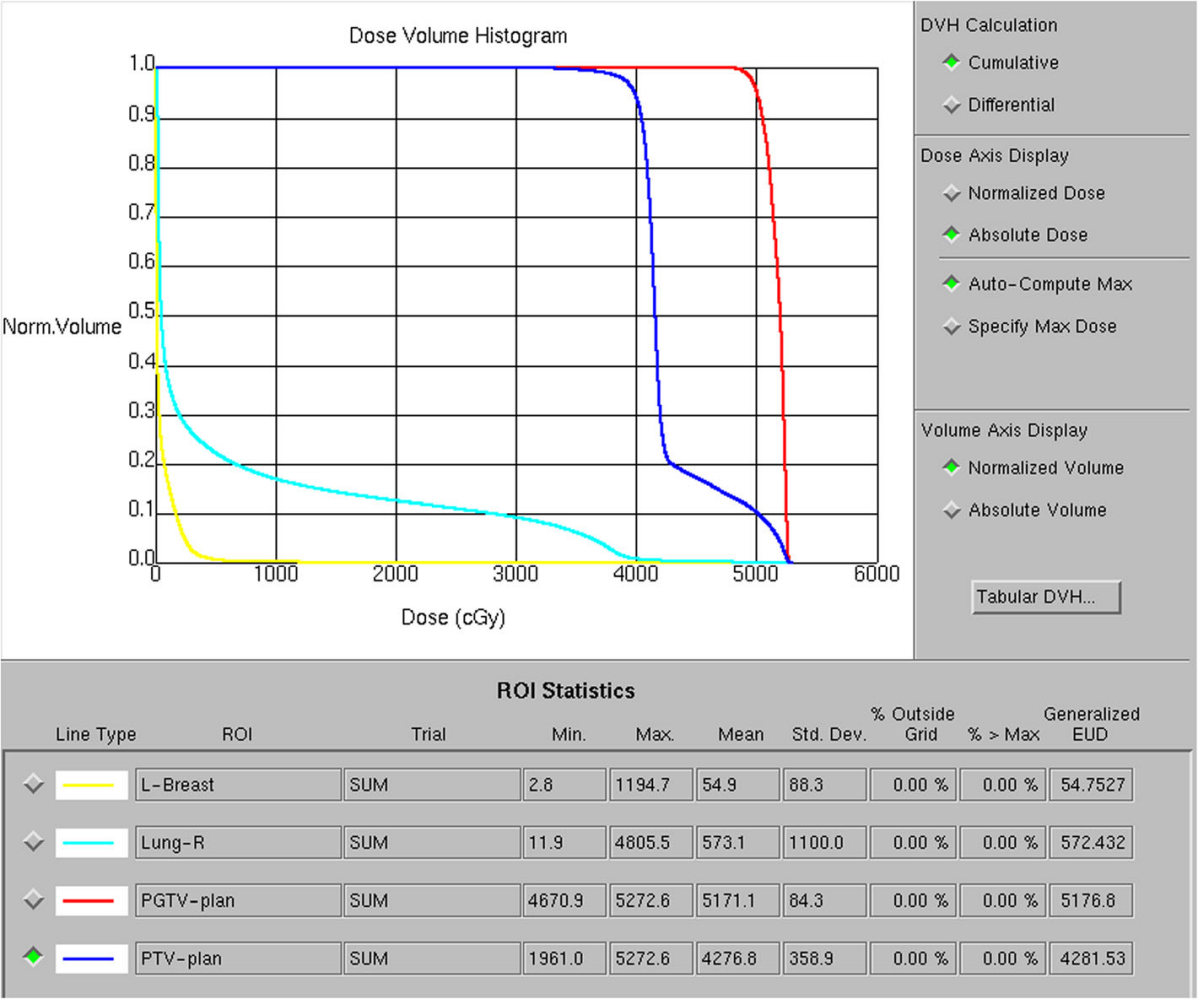
The dose volume histogram of organs at risk and target volume using simple intensity-modulated radiotherapy

**Fig. 6:**
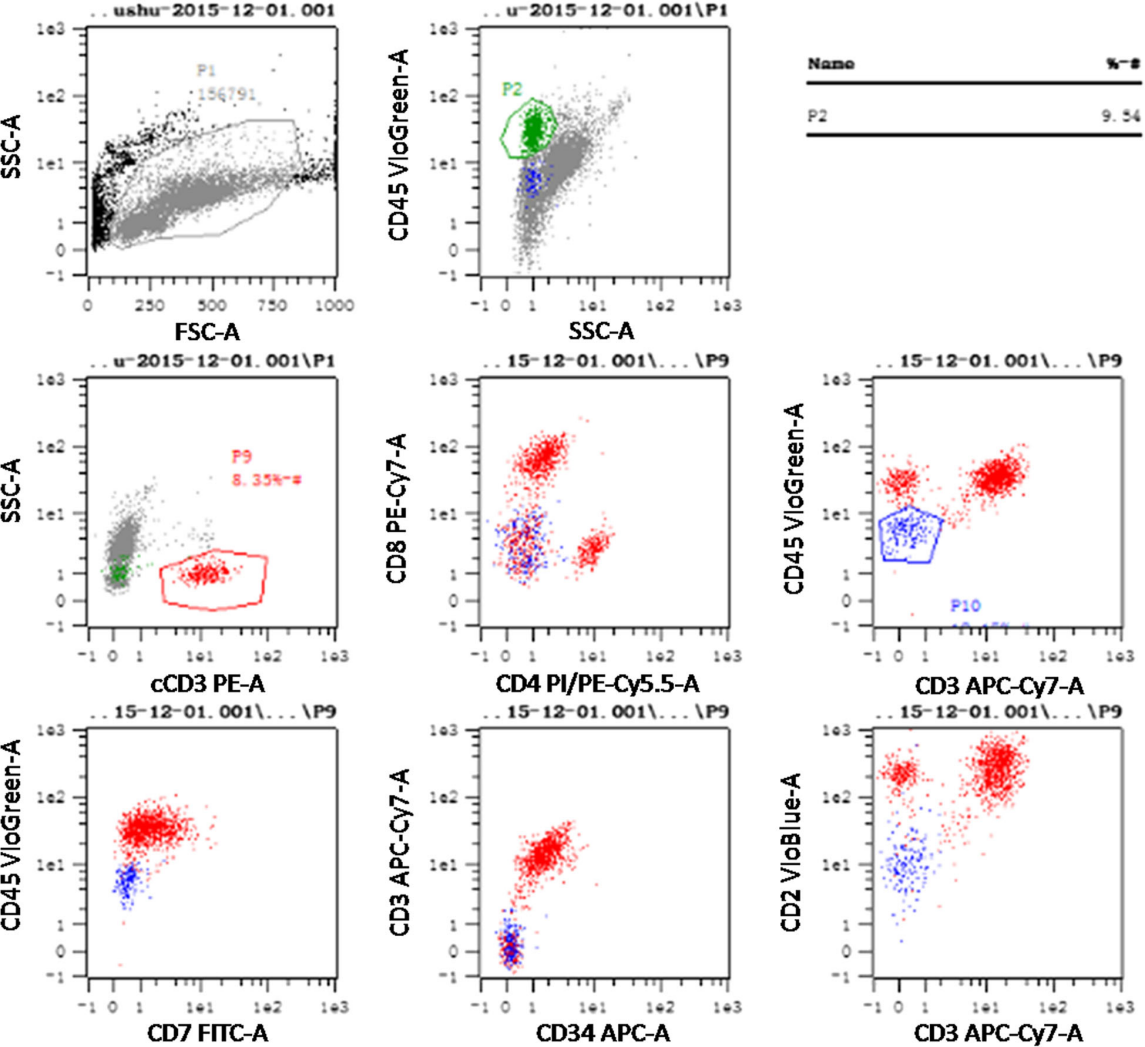
Flow cytometry results of the bone marrow aspirate obtained at the last follow-up (December 1, 2015) indicated complete remission

Six months after HSCT, the patient complained of a hard lump in the inner upper quadrant of the right breast. A subsequent breast ultrasound demonstrated two masses in her right breast. The larger one, located in the inner upper quadrant, measured approximately 35 mm × 26 mm × 10 mm with high vascularity and a Breast Imaging Reporting and Data System score of 4. The smaller one, a 9 mm × 4.3 mm hypoechoic nodule, was located at a 9 o’clock position. The biopsy of the large lump indicated the presence of a small round cell tumor (Fig. 2) with an immunohistochemistry profile similar to that of non-Hodgkin lymphoma: CD20 (-), CD3 (+), CD56 (+), CK (-), P63 (-), Syn (-), TTF-1 (-), and Ki-67 (+60 %). Bone marrow and cerebrospinal fluid evaluation revealed no evidence of leukemia. *SET-CAN* expression was negative. Therefore, a diagnosis of breast IEMR was established. After two cycles of chemotherapy with nelarabine (1500 mg/m^2^, 2310 mg d1, 3, 5, q28d) and one cycle of chemotherapy with VCP (vincristine, cyclophosphamide, and prednisone) -VP (vincristine, and prednisone) -VCP -VP, the large lump shrank to 15.6 mm × 5.5 mm. However, the small nodule showed no changes and caused pain. Positron emission tomography-computed tomography (PET/CT) demonstrated a moderately FDG (fluorodeoxyglucose) -avid nodule (standardized uptake value: 4.6) in the outer upper quadrant of the right breast and no focal uptake in the other sites (Fig. 3). Subsequently, the patient underwent simple intensity-modulated radiotherapy (sIMRT). The whole right breast received a dose of 40 Gy/2 Gy/20 f with a concurrent dose of 50 Gy/2.5 Gy/20 f (Fig. 4) to the FDG -avid lump in the outer upper quadrant. The dose volume histogram showed excellent dose coverage to the target volume and minimal dose to the surrounding normal tissue (Fig. 5). After irradiation of 14 f, the patient did not experience pain, and the lump in the outer upper quadrant disappeared. Furthermore, intensive evaluation of the bone marrow including cytologic, flow cytometry, and molecular examination revealed CR. The post-radiotherapy breast ultrasound also confirmed that the FDG -avid lump disappeared. Thus, radiotherapy resulted in CR with mild related side effects. Thereafter, the patient received four cycles of chemotherapy with decitabine (25 mg d1-5, q28d). The last evaluation of the bone marrow, conducted 18 months after HSCT, indicated CR (Fig. 6). The breast ultrasound conducted on April, 2016, 1 year after radiotherapy, still demonstrated CR.

## Discussion

The most common sites of extramedullary relapse (EMR) from ALL are the testis and central nervous system [9]. IEMR of the breast in ALL after allogeneic HSCT is extremely rare. According to the reports by Bekassy et al*.* [10], the incidence of breast relapse after HSCT in leukemia was 0.09 %. IEMR in the breast usually manifests as bilateral multiple nodules without specific radiologic characteristics [7]. In our case, involvement of only the right breast was an uncommon finding.

The most essential explanation is the protective graft-versus-leukemia effect on the bone marrow [11]. However, for extramedullary sites such as the central nervous system and testis, the effect of chemotherapy is compromised owing to the blood-brain or -testis barrier. Besides, allogeneic T cells can hardly reach these locations [12]. Philadelphia positive ALL is considered a high-risk indicator of relapse and dismal prognosis [7]. Horowitz et al*.* pointed out that the presence of GVHD was associated with a relatively lower risk of relapse in ALL after allogeneic HSCT [13]. Our patient did not develop GVHD, partially illustrating the occurrence of breast IEMR. Additionally, individuals who developed EMR before HSCT had a much higher risk of relapse in extramedullary site [14, 15]. Another explanation for IEMR is that blast cells have a high affinity to extramedullary locations because of specific adhesion molecules or receptors [6].

PET/CT was valuable in detecting EMR after HSCT even in uncommon sites [16]. In terms of a sensitive, whole-body imaging modality, PET/CT was helpful in accurate staging and evaluation of leukemia burden. As described in our case, PET/CT demonstrated that the larger lesion disappeared after chemotherapy, while the smaller one was still FDG -avid. Therefore, further intervention should be initiated to improve local control. The application of PET/CT is encouraged in cases of EMR after HSCT for accurate disease staging, delineating treatment strategies, and response evaluation.

*SET-CAN* fusion gene was first described in 1992 by von Lindern M et al*.* [17, 18] in a case of acute undifferentiated leukemia. *SET-CAN* positivity generally indicates resistance to chemotherapy, especially to high dose of glucocorticoids [19]. Van Vlierberghe P et al*.* pointed out *SET-CAN* boosted the progression of ATLL by activation of *HOXA* and inhibition of T cell differentiation [20]. Besides, *SET-CAN* is a valuable index in the minimal residual disease (MRD) monitoring for its high sensitivity. *SET-CAN* was positive at first visit in our patient and turned to negative when she suffered breast relapse, indicating that the bone marrow was CR and the breast relapse was EMR.

In comparison with its B-lineage ALL counterpart, IEMR in the breast in ATLL after HSCT is exceedingly rare. Howrey et al*.* described the case of a 15-year-old girl who received an umbilical cord blood transplant for refractory, relapsed T-cell ALL, and developed breast IEMR on day +373 after transplantation. Despite local radiotherapy to the breast mass, bone marrow relapse occurred over the next several weeks and the patient eventually died of progressive disease on day 140 after breast IEMR [21]. Firas et al*.* described the case of a 27-year-old woman with common T-ALL with a normal karyotype who developed IEMR in the left breast and left axillary lymph nodes on day +345 after allogeneic HSCT. Local radiotherapy helped achieve CR. However, on day +519 after HSCT, she experienced myocardial infiltration, and subsequent systemic chemotherapy induced CR. On day +707 after HSCT, relapse in the kidneys and bone marrow occurred. The patient died shortly thereafter [2]. In our patient, the HSCT was effective (CR at least 18 months after HSCT). However, long-term surveillance should be performed to monitor treatment outcomes.

Even though patients with EMR due to leukemia after allogeneic HSCT usually demonstrate a dismal prognosis, frequently followed by multiple relapses, they tend to show long-term survival [10, 15]. The optimal treatment remains uncertain owing to the rarity of IEMR after HSCT and the lack of large-scale retrospective studies [2]. Multiple factors such as the interval between IEMR and HSCT, patient age, performance status, and prior treatment strategy must be taken into consideration before a definite therapeutic algorithm is established [10, 15, 22, 23]. In the published cases, cases of IEMR in the breast demonstrated varying sensitivity to radiotherapy. In the study by Fadilah et al*.*, a patient with B-precursor ALL presented with bilateral breast and ovarian recurrence on day +172 after HSCT. Radiotherapy of 40 Gy to the breasts resulted in only partial remission. Subsequent chemotherapy with mitoxantrone, cytosine, and arabinoside and infusion of donor lymphocytes contributed to CR. Moreover, multidisciplinary intervention achieved at least 10 months of disease-free survival [5]. However, in the case presented by Conter et al*.*, the breast IEMR showed strong radiosensitivity at 30 Gy/15 f plus mild chemotherapy, which brought about rapid disappearance of the tumor [24]. In the case described by Firas et al*.*, 12 fractions of radiotherapy to the 10 cm tumor in the left breast and 3 cm left axillary lymph node helped achieve CR [2]. Radiotherapy might play an essential role in local control of breast IEMR. One study reported that the lack of radiotherapy at the first relapse in the right breast in a patient with ALL following allogeneic HSCT resulted in a second relapse in the same breast [25]. In our case, the two lesions in the right breast showed great heterogeneity. The larger one responded to chemotherapy well while the smaller one did not. The smaller lesion was resistant to chemotherapy but radiosensitive. This heterogeneity emphasized the importance of combined modality treatment in the elimination of leukemia cells to the greatest extent.

## Conclusions

The breast is a potentially involved site of relapse for ALL patients after HSCT even without evidence of bone marrow recurrence. For newly developed breast lumps in ALL individuals after HSCT, we should consider the possibility of local relapse despite bone marrow remission. Close follow-up is critical because early detection of tumor progression and timely intervention can guarantee a better prognosis. This case is novel in that it is rare and because it illustrates the value of PET/CT in disease evaluation and determining the best intervention. In future, multicentric clinical trials should be conducted to establish a consensus regarding this unique disease.

## Abbreviations

ALL: Acute lymphoblastic leukemia
ATLL: Acute T lymphoblastic leukemia
BU/CY: Busulfan/ cyclophosphamide
CAM: Cyclophosphamide, cytarabine and 6-mercaptopurine
CR: Complete remission
CSA: Cyclosporin
EMR: Extramedullary relapse
FDG: Fluorodeoxyglucose
GVHD: Graft-versus-host disease
HD-MTX: High dose methotrexate
HLA: Human leukocyte antigen
HSCT: Hematopoietic stem cell transplantation
IEMR: Isolated extramedullary relapse
MRD: Minimal residual disease
MTX: Methotrexate
PET/CT: Positron emission tomography-computed tomography
sIMRT: Simple intensity-modulated radiotherapy
VCP: Vincristine, cyclophosphamide, and prednisone
VDCLP: Vincristine, daunorubicin, cyclophosphamide, L-asparaginase and prednisone
VP: Vincristine, and prednisone
